# Case Report and literature review: X-linked severe combined immunodeficiency complicated by *Talaromyces marneffei* infection caused by a novel pathogenic *IL2RG* mutation

**DOI:** 10.3389/fped.2026.1769503

**Published:** 2026-04-22

**Authors:** Di Qing, Lin Lin, Fang Chen, Junzheng Peng

**Affiliations:** Department of Respiration, Guangzhou Women and Children's Medical Centre, Guangzhou Medical University, Guangzhou, Guangdong, China

**Keywords:** genetic test, IL2RG gene mutation, novel mutation, *Talaromyces* *marneffei*, X-linked severe combined immunodeficiency (X-SCID)

## Abstract

**Background:**

X-linked severe combined immunodeficiency (X-SCID), caused by mutations in the gamma-chain gene of the interleukin-2 receptor (*IL2RG*), is a prevalent form of SCID characterized by recurrent and fatal opportunistic infections that occur early in life. *Talaromyces marneffei* (*T. marneffei*) infection rarely occurs in children and has a high mortality rate.

**Case presentation:**

The patient was a 7-month-old male infant who presented with recurrent cough, fever, and hepatosplenomegaly. Lymphocyte subset analysis confirmed the presence of T-B + natural killer immunodeficiency, and blood culture was positive for *T. marneffei.* Whole-exome sequencing revealed a novel microdeletion insertion mutation (c.818_819delins A (p. Ile273Lys fsTer21) in *IL2RG*, resulting in a rare shift in the amino acid sequence of the coding protein. The child was diagnosed with X-SCID due to a novel *IL2RG* mutation, which was further complicated by *T. marneffei* infection. Despite receiving systemic anti-infection treatment, the patient died 3 days after discharge. To the best of our knowledge, this novel *IL2RG* mutation has not been reported previously.

**Conclusions:**

For early-onset *T. marneffei* infection, clinicians must maintain a high index of suspicion for underlying inborn errors of immunity, and definitive diagnosis hinges on genetic testing.

## 1 Introduction

Severe combined immunodeficiency (SCID) is a condition caused by gene pathogenic variation. The prevalence of SCID varies from approximately 1:50000 to 1:100000 (1). It can be divided into X-linked and autosomal recessive inheritance, with X-SCID as the most common form (2). X-SCID mainly presents with a severe decrease in T-cell count, with or without a decrease in natural killer (NK) cells and functional defects in B cells (3). Clinical manifestations include recurrent and life-threatening severe infections (4).

IL2RG is one of the three components of the interleukin-2 receptor common gamma chain (IL-2R) and a common component of various cytokine receptors, such as IL-4 and IL-7, which play key roles in T-cell and NK-cell maturation (5, 6).

In this study, we report the case of an infant with a novel pathogenic *IL2RG* and complicated by *T. marneffei* infection. Although the patient eventually died, this case still provides important clinical insights.

## 2 Case description

### 2.1 Chief complaint and admission history

**Figure 1:**
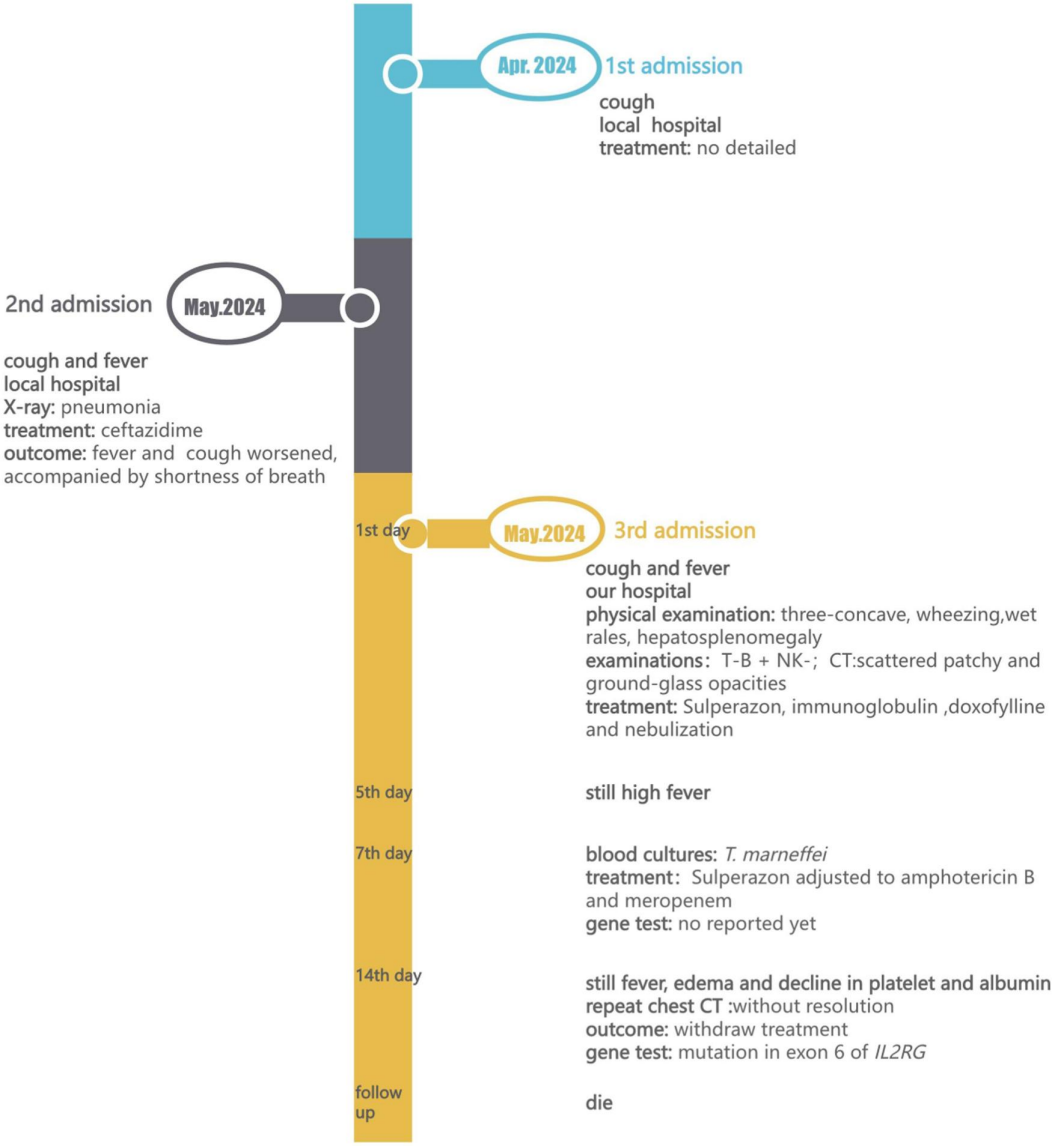
Timeline with chronological data; T, T cell; B, B cell; NK, Natural Killer cell; CT, Computed Tomography.

A 7-month-old infant, who had a history of recurrent cough and fever for 12 days, was admitted to our hospital in May 2024 (for the timeline, see Figure 1). In his previous admissions in local hospital, chest x-ray imaging revealed pneumonia, and he was prescribed ceftazidime. However, the fever did not improve, and the cough worsened, accompanied by wheezing and shortness of breath.

### 2.2 Past medical and family history

He also had a history of pneumonia 1 month prior. His birth and personal history were unremarkable.

### 2.3 Physical examination

On admission, he had a weight of 7.5 kg (P3 = 7.5 kg), height of 72 cm (P50 = 70 cm), temperature of 37.8 °C, heart rate of 120 beats per minute, respiratory rate of 40 beats per minute, blood pressure of 88/56 mmHg, and oxygen saturation (SpO2) of 98% (mask oxygen inhalation). The presence of the three-concave sign, wheezing, and bilateral wet rales suggested respiratory distress. Furthermore, the abdomen was soft on palpation, and the liver was palpable 2 cm below the costal margin, with smooth and soft edges, consistent with normal findings.

### 2.4 Laboratory examinations

Laboratory tests indicated an abnormally low white blood cell count of 3.13 × 10^9^/L (4.4–11.9 × 10^9^/L), normal platelet count of 121 × 10^9^/L (100–472 × 10^9^/L), and low hemoglobin level of 78 (112–149 g/L), C-reactive protein of 16.4 (0–5) mg/L, and pro-calcitonin of 1.63 ng/mL. The immunological assessment revealed a significant decrease in IgA, IgM, T lymphocytes and NK cells (Table 1). Respiratory etiological examination was positive for respiratory syncytial virus, rhinovirus, and parainfluenza virus.

**Table 1:** Laboratory findings of the patient on the day of admission.

Laboratory index	Result	Reference range
IgG (g/L)	0.69 ↓	3.3–8.8
IgA (g/L)	<0.1 ↓	0.11–0.76
IgM (g/L)	0.14 ↓	0.33–1.25
CD4 + T-cell (cells/µL)	18.54 ↓	410–1590
CD4 + T-cell/lymphocyte (%)	1.84 ↓	30–60
CD8 + T-cell (cells/µL)	73.74 ↓	190–1140
CD8 + T-cell/lymphocyte (%)	7.32 ↓	13–41
B cell (cells/µL)	810.96 ↑	90–660
B cell/lymphocyte (%)	84.59 ↑	5–18
NK (cells/µL)	27.01 ↓	90–590
NK/lymphocyte (%)	2.85 ↓	7–40
PBS	68.60 ↑	<10
PMA	99.90	>90
SI	95.28↓	>100

NK, natural killer; PBS, phosphate buffered saline; PMA, phorbol 12-myristate 13-acetate; SI, stimulus index.

### 2.5 Radiological findings

**Figure 2:**
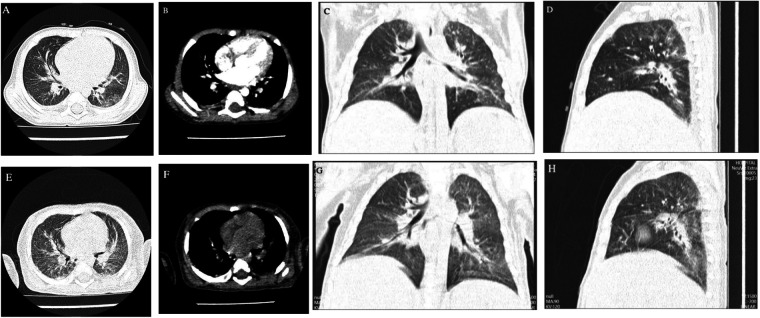
Uneven aeration in both lungs, with scattered patchy and ground-glass opacities, accompanied by bronchial wall thickening. **(A-D)** was the HRCT at admission; **(E-H)** was the HRCT 14 days after hospitalization.

Abdominal ultrasonography showed liver and spleen enlargement and dilation of left renal pelvis. High-resolution computed tomography of the chest revealed uneven aeration in both lungs, with scattered patchy and ground-glass opacities, accompanied by bronchial wall thickening (Figures 2A–D).

### 2.6 Therapeutic intervention

By day 5 of admission, the patient continued to have high fever persisted up to 40 °C; normothermia was not achieved despite combination therapy, including Sulperazon as antibiotic treatment, immunoglobulin for supportive care, doxofylline for asthma relief, and nebulization. On day 7, blood cultures yielded *T. marneffei*, and the treatment regimen was adjusted to include amphotericin B and meropenem.

Lymphocyte subset analysis revealed low T and NK lymphocyte counts, alongside increased B lymphocytes, confirming T-B + NK -immunodeficiency. Therefore, the patient and his parents underwent genetic testing to identify the molecular defects and genetic risk of immunodeficiency. Whole-exome sequencing identified a delins mutation in exon 6 of *IL2RG* (chrX: 70328484 −70328485), resulting in a frameshift mutation at the amino acid level of the coding protein of *IL2RG* (NM_000206.3), c.818_819delins A (p. Ile273Lys fsTer21). Experimental data indicated that the patient inherited the mutation from his mother, who was heterozygous, whereas the father was not a carrier. Next-generation sequencing of the patient's brother identified no mutation, consistent with a wild-type genotype.

### 2.7 Outcomes

The patient was ultimately diagnosed with SCID complicated by *T. marneffei* infection. By day 14 of admission, the patient continued to have high fever and a poor general condition, accompanied with marked edema and progressive decline in platelet count and albumin level. Repeat chest CT showed pre-existing exudates without significant interval resolution and a new infectious focus in the right middle lobe (Figures 2E–H). His parents elected to withdraw treatment and proceed with discharge. The child died 1 week later during follow-up.

## 3 Literature review

A literature search was performed across PubMed, Web of Science, ClinVar, Embase, and Medline using the search terms “*IL2RG*,” “*T. marneffei*,” “Penicillium marneffei,” and “SCID.” This approach ensures a broad coverage of relevant literature, as PubMed encompasses Medline and includes a wider range of data types, and Web of Science and Embase provide additional sources for comprehensive research. This search yielded four reports (7–10) of five children with X-SCID complicated with *T. marneffei* infection caused by *IL2RG* mutation, published before October 2025 (Table 2). The six children, including the current case, were male, with most ages ranging from 3 to 8 months. All patients presented with fever, cough, and hepatosplenomegaly. Additionally, most patients had skin lesion (*n* = 4), diarrhea (*n* = 3), ascites (*n* = 1), and edema (*n* = 1). Among the six children, four were initially treated with amphotericin B, an antifungal; regrettably, three of them succumbed to infection.

**Table 2:** Clinical manifestations of *IL2RG* mutation in children with *T. marneffei* infection.

Patients	Age (months)	GenderSex	Nucleotide variation	Types of gene mutation	Mutation source	Protein consequence	Clinical features	Treatments	Outcomes
P1 (7)	3	Male	c.185G > A	Missense mutation	Maternal	p.Cys62Tyr	fFever, diarrhea, dyspnea, skin lesion, and hepatosplenomegaly	Caspofungin and amphotericin B liposomes	Died
P2 (8)	8	Male	c.464G > A	Nonsense mutation	Maternal	p.Trp155X	fFever, cough, diarrhea, malnutrition, weight loss, skin lesion, and hepatosplenomegaly	Amphotericin B liposomes and itraconazole	Improvement
P3 (8)	4	Male	c.464G > A	Nonsense mutation	Maternal	p.Trp155X	Fever, cough, dyspnea, malnutrition, weight loss, skin lesion, lymphadenopathy, hepatosplenomegaly, and ascites	vVoriconazole and itraconazole	Died
P4 (9)	8	Male	No mention	No mention	No mention	No mention	Fever, cough, anemia, rash, hepatosplenomegaly, and hematuria	Amphotericin B, voriconazole, and itraconazole	No follow-up
P5 (10)	164	Male	No mention	No mention	No mention	No mention	Fever, cough, diarrhea, hepatosplenomegaly, and superficial lymphadenopathy	Voriconazole	Improvement
P6 (our case)	7	Male	c.818 > A	Nonsense mutation	Maternal	p.Ile273Lys fsTer21	Fever, cough, hepatosplenomegaly, and edema	mphotericin B and meropenem	Died

## 4 Discussion

This report presents a case of a 7-month-old male infant diagnosed with *T. marneffei* infection and X-SCID caused by a novel and rare frameshift mutation in *IL2RG*, which was identified through whole-exome sequencing. Moreover, the clinical characteristics and genetic findings from previously reported cases of children with SCID caused by *IL2RG* mutations complicated by *T. marneffei* infection were reviewed and summarized.

*T. marneffei* infection is a rare opportunistic infection, predominantly occurring in Southeast Asia and Southern China. Clinical features of *T. marneffei* infection in HIV-negative children mainly include fever, weight loss, lymphadenopathy, and other symptoms (11, 12). This study also retrospectively analyzed the clinical data of six pediatric patients with non–HIV-associated *T. marneffei* infection. All patients were male, which were predominantly infants who presented with nonspecific clinical manifestations such as fever, cough, superficial lymphadenopathy, hepatosplenomegaly, and elevated serological inflammatory indicators.

Infection intensity and prognosis are closely related to the host's immune status (13, 14); as mentioned earlier, most *IL2RG* pathogenic mutations lead to severe T-cell defects, further causing typical SCID characterized by T-B + NK- immunodeficiency. Patients with *STAT1* mutations are also susceptible to *T. marneffei* infection, but through distinct mechanisms. *STAT1* mutations directly abrogate interferon-*γ* signaling and cause a selective defect in antifungal immunity, who presente with infections predominantly caused by fungi and mycobacteria and showed no overt impairment in T-cell development and did not exhibit signs consistent with severe combined immunodeficiency. Among the six patients with *IL2RG* mutations, three ultimately succumbed. However, atypical X-SCID can manifest as T + B + NK- immunodeficiency with milder immune deficiency, as in case 5 with a late onset and favorable prognosis, which is considered related to the presence of a certain number of T cells. In this patient, T-cell immune typing analysis was not conducted; therefore, we failed to perform an in-depth assessment of the differentiation and function of immature and memory T-cell subsets, which is crucial for understanding immune dysfunction (15).

Current treatment is based on long-term consolidation and maintenance treatment with itraconazole after 2 weeks of highly effective amphotericin B induction therapy (16, 17). Voriconazole and itraconazole are also prescribed in the treatment of TM in patients without HIV infection, and their safety and efficacy have been confirmed (18). Of the six patients, four were administered amphotericin. P5 presented with an older age at onset and mild clinical manifestations, achieved clinical improvement, and was discharged after receiving voriconazole monotherapy. In patients with inborn errors of immunity (IEI) complicated by non–HIV-associated *T. marneffei* infection, the total treatment course is typically longer than that in patients with HIV-associated *T. marneffei* infection, and some cases require lifelong therapy (19). In addition to pharmaceutical treatment, hematopoietic stem cell transplantation is the primary method of immune reconstitution, followed by gene therapy (20). Despite standardized anti-infective therapy, the patient did not have the opportunity for stem cell transplantation.

**Figure 3:**
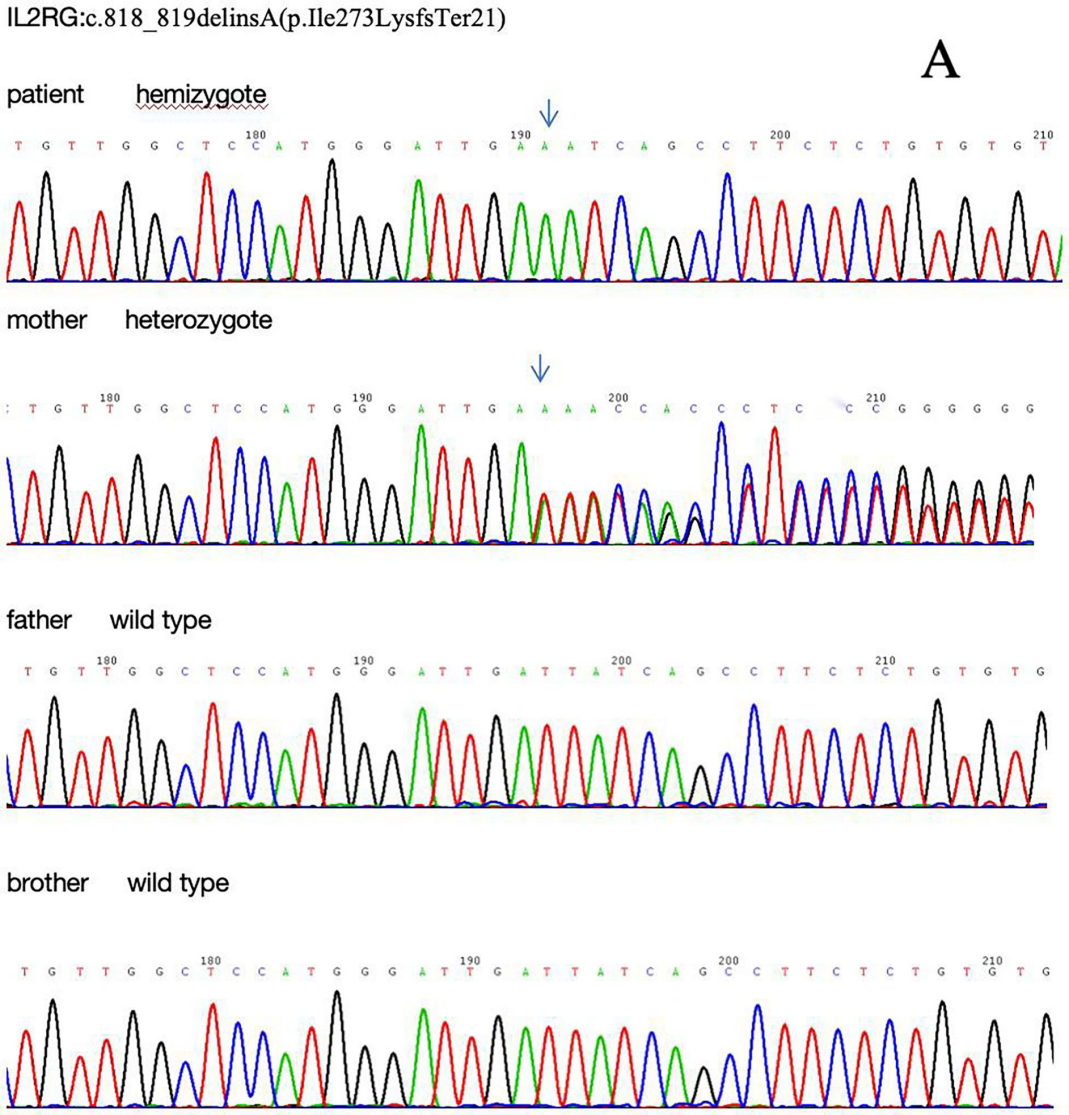
*IL2RG* mutation detected in the present case. **(A)** Sanger chromatogram. **(B)** shows the genomic structure of the *IL2RG* gene: B1 depicts the chromosomal organization of the gene, with black bars representing exons (coding regions) and white/grey bars representing introns (non-coding regions); B2: panel zooms in on the gene's linear structure, highlighting the positions of exons 1–8 and marking the location of a mutation within exon 6. B3-4: provides a detailed view of the DNA sequence spanning exons 5–6, along with its corresponding amino acid translation, and a magnified inset focusing on the mutated codon in exon 6. **(C)** Schematic diagram of the mutation protein.

In the present case, we detected a novel frameshift mutation that has not yet been reported. A frameshift mutation in exon 6 of *IL2RG* on the X chromosome converts isoleucine at position 273 of the encoded protein to lysine, resulting in the introduction of a premature termination codon (Figure 3), which met the PVS1 criterion. In the present case, the patient's mother was heterozygous with a normal phenotype, whereas the father and elder brother were wild-type, satisfying the PM2_Supporting criterion and consistent with the inheritance pattern of an X-linked recessive genetic disorder. According to the guidelines of the American College of Medical Genetics and Genomics, combined with the clinical phenotype and results of family genetic analysis of the patient, this mutation meets the criteria for PVS1 + PM2 pathogenicity classification and is considered a likely pathogenic mutation (21, 22, 23). SCID, given its early onset and high mortality rate, has been prioritized as the primary IEI for inclusion in newborn screening (NBS). To date, more than 20 countries have laid a technical foundation for NBS through quantitative detection of T-cell receptor excision circles and kappa-recombination excision circles. The implementation of this screening is expected in China in the near future.

**Figure:**
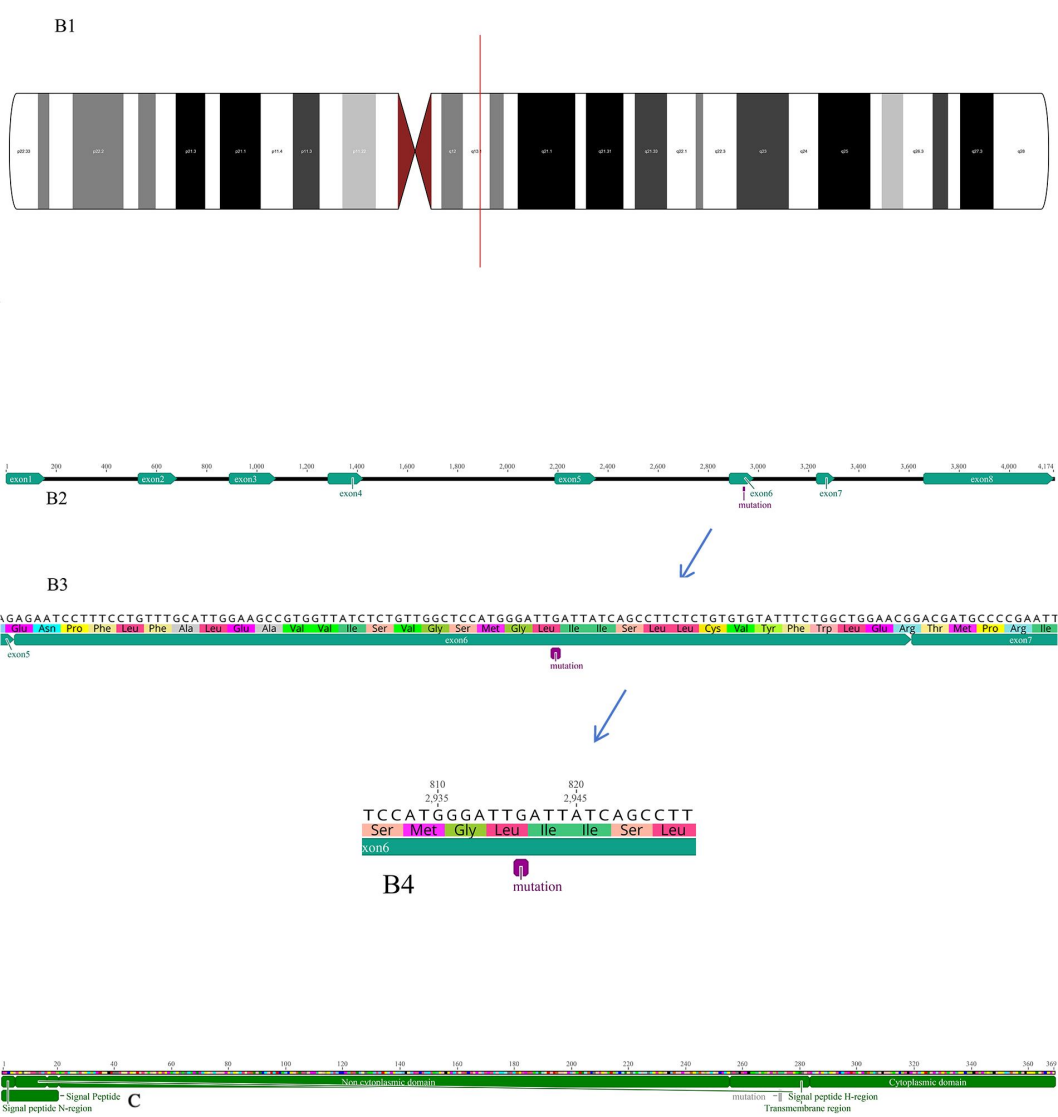


## 5 Conclusion

X-SCID resulting from *IL2RG* mutations is a rare immunological disorder associated with severe or unusual infections in infants, and genetic analysis is essential to maintain the optimal therapeutic window. These patients must be assessed for potential opportunistic pathogens and administered a full course of anti-infective therapy.

## Data Availability

The datasets presented in this study can be found in online repositories. The names of the repository/repositories and accession number(s) can be found in the article/Supplementary Material.
